# Oneida County Veterinary Medical Association

**Published:** 1896-09

**Authors:** J. M. Currie

**Affiliations:** Secretary


Secretary.

### ONEIDA COUNTY VETERINARY MEDICAL ASSOCIATION.

The regular quarterly meeting was called to order in the parlors of Stanwix Hall, Rome, N. Y., at 1.30 P.M., August 4, 1896, by Dr. F. Morrow, President. The following members responded to roll-call: Drs. F. Morrow, Oneida; L. G. Moore, Trenton; H. W. Skerritt, Deansboro; R. C. Hurlburt, Boonville; Wilson Huff, Rome; and J. M. Currie, Rome. The minutes of the last meeting were read and approved.

Dr. H. W. Skerritt presented the names of Drs. H. D. Stebbins, West Winfield, and R. M. Weightman, Waterville, for membership. They were reported favorably upon by the Board of Censors and declared elected. The gentlemen being in waiting, they were then introduced.

After the regular routine of business came the reading of papers, etc.

Dr. R. C. Hurlburt read a very interesting paper on Equine Dentistry. In the discussion which followed, the travelling dentist was severely scored for making such rash promises to his clients and failing to fulfil the same. It was asserted that veterinarians as a class receive abuse which the travelling dentist alone should bear. This paper was fully discussed, and the writer was tendered a vote of thanks for his valuable contribution; after which a general and interesting talk took place on such subjects as "Tetanus and its Treatment with Gelsemium," "Iodine Treatment in Purpura Hemorrhagica," "Barium Chloride in Intestinal Difficulties." Other interesting items were presented.

Dr. Moore was appointed as essayist for the next meeting, to be held on the first Tuesday in November. There being no further business, the meeting then adjourned.

J. M. Currie, V.S.,
Secretary.

